# Exploring global calcimimetics research trends: a systematic and thematic review of Web of Science and Scopus databases from 1997 to 2024

**DOI:** 10.3389/fneph.2025.1617466

**Published:** 2025-08-13

**Authors:** Riad Abdelrahman, Taha H. Musa, Chiamaka Linda Mgbechidinma, Eltieb Omer Ahmed

**Affiliations:** ^1^ School of Medical and Health Sciences, Faculty of Pharmacy, Libyan International Medical University, Benghazi, Libya; ^2^ Faculty of Graduate Studies and Scientific Research, National Ribat University, Khartoum, Sudan; ^3^ School of Life Sciences, Centre for Cell and Development Biology and State Key Laboratory of Agrobiotechnology, The Chinese University of Hong Kong, Hong Kong, Hong Kong SAR, China; ^4^ Department of Microbiology, University of Ibadan, Ibadan, Oyo, Nigeria; ^5^ Department of Pharmacy Practice, Faculty of Pharmacy, International University of Africa, Khartoum, Sudan

**Keywords:** a systematic and thematic, calcimimetics, chronic kidney disease, diabetic, scopus, Web of Science

## Abstract

**Background:**

Calcimimetics are a group of medications that increase the sensitivity of the calcium receptors to extracellular calcium ions and inhibit the release of parathyroid hormone (PTH) in patients with chronic kidney disease (CKD).

**Objectives:**

The aim of this study was to analyze the global trends in the publication of articles on calcimimetics through bibliometric analysis of the Web of Science and Scopus databases, as well as to identify the most highly cited articles from 1997 to 2024.

**Methods:**

Systematic and thematic analyses were performed to provide substantial insights into calcimimetic research. Data were analyzed using VOS viewer (var1.6.6) and the Biblioshiny tool.

**Results:**

A total of 3,500 documents were identified for analysis. There was an exponential growth in calcimimetic-associated publications (from 57 documents in 2004 to 258 in 2021). The mean of the total citations per article showed a decrease from 226 in 1998 to 0 in 2024. The United States was the most productive country. Goodman W. emerged as the most prolific author, with high-level metrics [*n* = 45, total number of citations (TNC) = 4,768, *h*_index = 27]. Fukazawa M. showed the longest research activity in the field, with 97 published documents in 25 years. Nephrology Dialysis Transplantation was the most published journal, with 112 documents and with an *h*_index of 43. The thematic KeyWords Plus analysis identified three key domains, including pharmacological targets (CaSR and cinacalcet) reported in niche themes and central CKD and mineral bone disorder (MBD) pathway (hemodialysis, vascular calcification, and vitamin D) case reports in emerging/declining themes. The small correlation between “diabetes” and “mineral metabolism” (despite shared CKD complications) suggests a critical research gap. While our thematic map highlighted robust research on the pathophysiology of CKD-MBD, critical clinical outcomes remain underexplored. Future trials should highlight these gaps, particularly in high-risk subgroups such as diabetic patients with CKD.

**Conclusion:**

The results of this review offer a summary of the global landscape, the key research areas, and possible future directions in calcimimetic research. This information can assist researchers in exploring the knowledge structure and understanding future trends in calcimimetic research, as well as in supporting collaboration toward advanced global research on calcimimetics.

## Introduction

Chronic kidney disease (CKD) is defined by abnormalities in the kidney structure or function that present for 3 months or longer. A new classification system was proposed by the Kidney Disease Improving Global Outcomes (KDIGO), called CGA staging (cause, GFR, and albuminuria), which incorporated the glomerular filtration rate (GFR) and urine albumin-to-creatinine ratio (1).

CKD affects approximately 10% of the general population worldwide, and diabetes and high blood pressure are the most common causes of the disease (2). CKD–mineral bone disorder (CKD-MBD) is a complex syndrome defined by the KDIGO as a term used to collectively describe the mineral [e.g., phosphorus, calcium, parathyroid hormone (PTH)], bone (osteodystrophy), and soft tissue calcification abnormalities that develop as a complication of CKD. MBDs represent a clinical condition that, when present and not sufficiently controlled, is predictive of a very high risk of death and cardiovascular (CV) events (3).

Secondary hyperparathyroidism (SHPT) is a common complication of CKD and is a component of CKD-MBD that results from hyperphosphatemia, hypocalcemia, and the trade-off between hyperparathyroidism, decreased production of active vitamin D, and resistance to vitamin D (4).

The management of CKD, generally, should be based on the KDIGO clinical guidelines. Pharmacologic management of PTH, phosphorus, and calcium balance is essential in preventing the development of SHPT and in slowing down CKD and its associated consequences (1, 5).

In patients with stage 5 CKD who require PTH-lowering therapy, KDIGO suggests calcimimetics, calcitriol, or vitamin D analogs, or a combination of calcimimetics with calcitriol or vitamin D analogs (6).

The discovery of extracellular calcium-sensing receptors (CaSR) has prompted research on calcimimetic agents that allosterically modulate CaSR. CaSR have been identified in the parathyroid gland, thyroid, nephron, brain, intestine, bone, lung, and other tissues.

Calcimimetic agents increase the sensitivity of CaSR to extracellular calcium ions and inhibit the release of PTH, lowering its levels within hours of administration (7).

A few examples of calcimimetics include cinacalcet, etelcalcetide, evocalcet, and upacicalcet (8).

The term “calcimimetics” was coined by Nemeth et al. in 1990 and appeared in the public domain at the annual meeting of the American Society of Bone Mineral Research in 1993. The definition of “calcimimetics” is broad, representing all types of ligands that mimic the effects of extracellular calcium (Ca^2+^) on CaSR and inhibit the secretion of PTH.

Nemeth et al. classified calcimimetics into two pharmacological types: type I and type II. Type I calcimimetics act as agonists on the parathyroid cell CaSR. They do not require the presence of extracellular Ca^2+^ to affect PTH secretion. On the other hand, type II calcimimetics have no effects on the PTH secretion at very low or high concentrations of extracellular Ca^2+^, but shift the concentration–response curve for extracellular Ca^2+^ to the left, thereby lowering the half maximal effective concentration (EC_50_) for extracellular Ca^2+^. This mode of action suggests that type II calcimimetics function as positive allosteric modulators of CaSR rather than simple agonists (9).

Calcimimetics reduce the serum levels of PTH and calcium, with a leftward shift in the set point for calcium-regulated PTH secretion. Calcimimetics are a potential alternative for patients contraindicated for parathyroidectomy (PTX) or those who had a failed previous PTX and have recurrent primary HPT. SHPT develops early in CKD and is present virtually in all patients with end-stage renal disease (ESRD). SHPT is a progressive disease and is associated with several systemic complications, including renal osteodystrophy, soft tissue and vascular calcifications, and adverse cardiovascular outcomes (10, 11).

In renal allograft recipients with tertiary HPT and hypercalcemia, calcimimetics comprise a promising treatment option to control the parameters of calcium phosphate metabolism and may be a valid alternative to PTX. Based on its unique mechanism of action, the calcimimetic cinacalcet may play a role in the medical treatment of the primary and tertiary forms of HPT, in addition to its registered indication for the treatment of SHPT.

One study revealed that calcimimetics have had no impact on the indications for surgery of tertiary hyperparathyroidism (12–14).

A lot of studies have investigated the use and effectiveness of calcimimetics in the management of electrolyte abnormalities in different diseases (15–18). The calcimimetic cinacalcet is the first agent in this class to be approved by the Food and Drug Administration (FDA) in March 2004 for the treatment of SHPT in end-stage kidney disease.

Etelcalcetide, another calcimimetic agent owned by Amgen and Ono Pharmaceuticals (Japan), was approved in February 2017. It is administered intravenously. While cinacalcet is an allosteric modulator of CaSR, etelcalcetide acts as a direct CaSR agonist (19). Cinacalcet has been demonstrated to be an effective agent for reducing and sustaining intact parathyroid hormone (iPTH) within target concentrations in hemodialysis (HD) patients. Cinacalcet offers an additional therapeutic option for lowering the iPTH when vitamin D cannot be increased due to elevated calcium or phosphorus. Cinacalcet is not FDA-approved for use in patients with CKD not receiving dialysis as it is associated with frequent hypocalcemic episodes (20). Nausea and vomiting are the most common adverse events associated with cinacalcet. Moreover, 66% of patients administered cinacalcet experience at least one episode of hypocalcemia (serum calcium <8.4 mg/dl) (21, 22).

Evocalcet, which has been approved by the Pharmaceutical Affairs Act in Japan, is another calcimimetic that has been proven effective in the management of SHPT, with lower incidence of gastrointestinal side effects compared with cinacalcet (23). Evocalcet has fewer gastrointestinal-related adverse events while suppressing PTH at a lower dose than cinacalcet. These data suggest that evocalcet may contribute to better adherence and sufficient dose escalation in patients with SHPT (24).

Upacicalcet is a novel SHPT drug that targets the amino acid binding site of CaSR. It is an intravenous calcimimetic agent developed by Sanwa Kagaku Kenkyusho (Nagoya, Japan) for the treatment of SHPT in patients undergoing HD. Upacicalcet received its first approval on June 23, 2021, in Japan (25, 26).

Over the past two decades, there has been an increased scholarly focus on calcimimetics in terms of quantitative data, but scarcely in qualitative aspects.

Africa, particularly Sub-Saharan Africa, faces major challenges with respect to CKD. There is an increasing prevalence due to the combined effects of hypertension, diabetes, and human immunodeficiency virus (HIV). Two systematic reviews that determined the prevalence of CKD in the general African population found a prevalence of 13.9% (27). African scholars need to be abreast of the latest management measures and updated guidelines for CKD treatment.

This study examines the implications of systematic and thematic trends through bibliometric analysis to assess the growth and evaluation of research across various scientific fields. The data from these studies were interpreted using various metrics to assess impacts and the performance indicators within research policies and the broader research ecosystem. Given the rising demand for these types of analyses, in particular among policymakers and funding agencies, they are becoming increasingly crucial for the evaluation of research productivity and impact within many scientific communities.

Bibliometric studies effectively track the overall research trends and analyze the connections between authors and research institutions. Therefore, this study aimed to examine the characteristics of the top articles on calcimimetics indexed in the Web of Science (WoS) and Scopus databases, employing both systematic and thematic analyses as approaches, which have gradually emerged as valuable methods in the professional community. The current investigation aimed to answer the following scientific inquiries.

In this review, we visualize the results obtained from the WoS and Scopus databases and summarize the research hotspots and development trends, as well as the research gaps. Building upon the practical consistency in exploring global trends in calcimimetic research using systematic and thematic analyses, this study examines the collection of comprehensive body of literature published over the past year. The goal was to contribute to the growing scientific body of evidence in the field of calcimimetics by tracking the growth of research on calcimimetics, understanding the key contributions of authors and the corresponding authorship countries, and exploring changes in the identification, analysis, and interpretation of the pattern themes, emerging topics, and authorship over time within qualitative data accessed in the field.

## Methods

### Sources of data

Metadata toward exploring global calcimimetic research trends were from the Scopus database (https://www.scopus.com/) and the WoS edition (Science Citation Index Expand (SCI-Expand; https://www.webofscience.com/wos/) database, which was originally produced by the Institute for Scientific Information and was later developed by Thomson Reuters and then Clarivate Analytics. The WoS is considered a premier academic research database that provides scholars globally access to a vast collection of high-quality, peer-reviewed scholarly literature across many disciplines, as well as for determining emerging trends of publications and impactful research.

Both the Scopus and WOSCC (Web of Science Core Collection) platforms enable unique advanced searches as two of the world’s most trusted citation indexes for scientific and scholarly research, providing researchers with comprehensive datasets used subsequently for bibliometric analysis across many disciplines.

**Figure 1:**
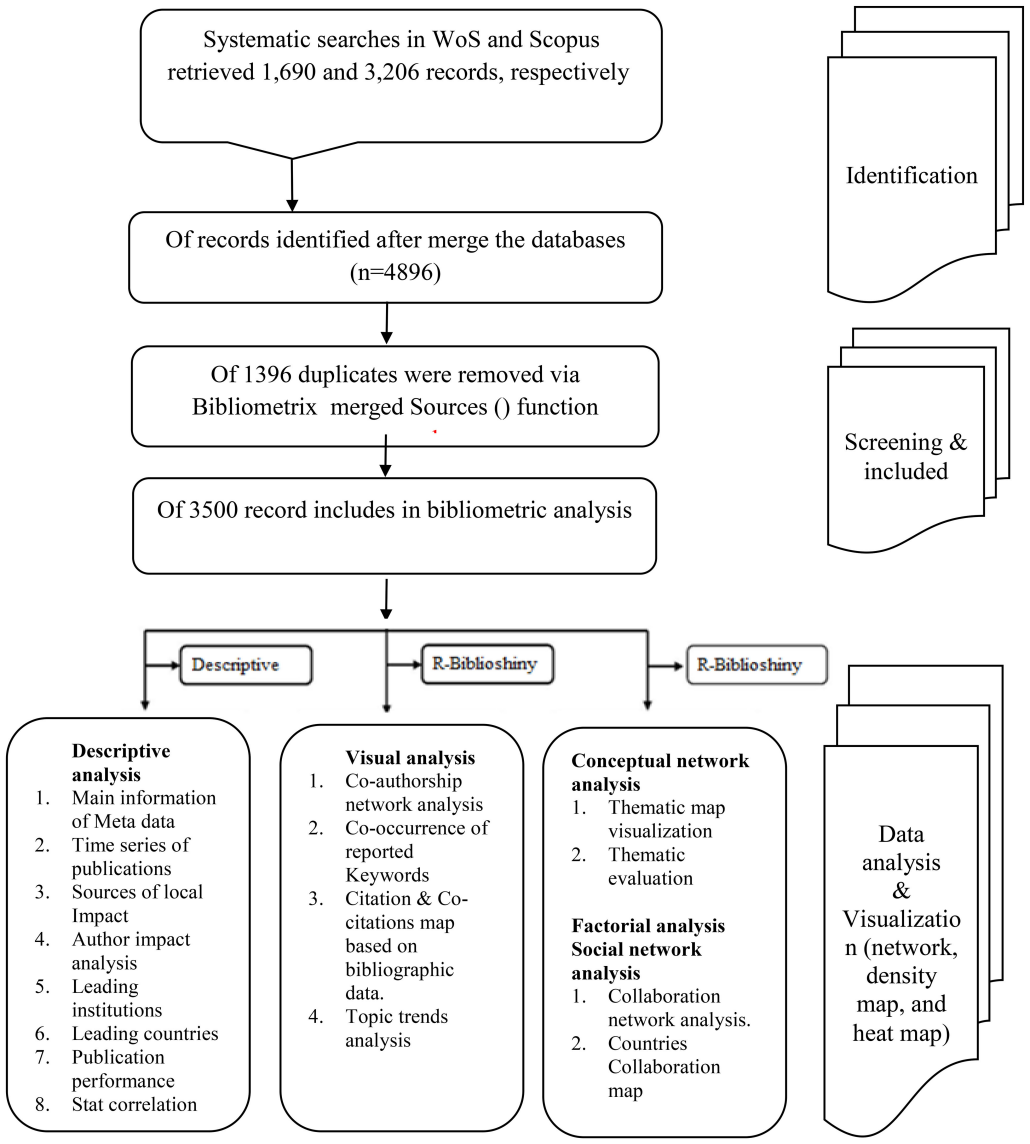
Flowchart of the search approach used in the data collection process from the Web of Science (WoS) and Scopus databases. Source: generated by the authors.

On March 28, 2025, we searched the WOSCC and Scopus databases for articles on global calcimimetic research in peer-reviewed journals indexed in the WOSCC database (SSCI and SCIE). The search strategy and a flowchart of article screening are presented in **Table 1** and **Figure 1**, respectively.

**Table 1:** Search strategy.

Database	Search terms	Filters	No. of documents
Web of Science (SSCI and SCIE)	(Calcimimetics* OR a calcimimetics* OR cinacalcet* OR evocalcet* OR etelcalcetide* OR upacicalcet*)	Document type (review OR article) and language (English)	1,690
Scopus	(Calcimimetics* OR a calcimimetics* OR cinacalcet* OR evocalcet* OR etelcalcetide* OR upacicalcet*), and the bibliographic data based on topic search using the query keywords in WoS database (Calcimimetics* OR a calcimimetics* OR cinacalcet* OR evocalcet* OR etelcalcetide* OR upacicalcet*)	Document type (review OR article) and language (English)	3,206

Subsequently, a Boolean search process was used, which included the following subject term search formula: keywords (K), Title (T), and Abstract (A) in the Scopus database related to spatial calcimimetics as (Calcimimetics* OR a calcimimetics* OR cinacalcet* OR evocalcet* OR etelcalcetide* OR upacicalcet*), as well as the bibliographic data based on the topic search using the query keywords in the WoS database (Calcimimetics* OR a calcimimetics* OR cinacalcet* OR evocalcet* OR etelcalcetide* OR upacicalcet*). The metadata on global calcimimetics were extracted by the authors on March 28, 2025, to avoid daily update bias as the database is still open and the average citations can increase per day. The databases used were selected based on data availability and accessibility.

The origin search record on global documents associated with calcimimetics indexed in WoS was 2,589 documents after filtering by document type (review OR article) and language (English), with 1,690 remaining articles from Science Citation Index Expand (SCI-EXPAND). In addition, the Scopus data resulted in 4,379 documents after filtering by document type (review OR article) and language (English), with 3,206 articles remaining.

The search was performed on March 28, 2025, which resulted in 4,896 documents from the Scopus and WoS databases. These were collected and exported in the following formats: as RIS, plain text, BibTeX, EndNote, and CSV in WoS and as RIS, BibTeX, CSV, EndNote, and XML in Scopus. The proposed analysis was set to screen calcimimetic-associated research, and the period was limited to the year 1990.

### Inclusion and exclusion criteria

Complete calcimimetic-associated publications from the WoS and Scopus databases were included in our analysis. The following types of reported documents were excluded from Scopus: letter, note, conference paper, short survey, editorial, book chapter, erratum, early access, proceeding paper, and undefined. Moreover non-English publications were excluded, such as those in Spanish, German, French, Russian, Portuguese, Czech, Polish, Slovenian, and Korean.

### Merging Scopus and the Web of Science databases

Reported clean data extracted by the authors from Scopus and WoS were merged into a single databases after removing duplicates using the bibliometrix analysis tool in RStudio (version R-4.4.3 for Windows). The merging process was performed according to the methods of previous scholars in the field of scientific research (1).

### Bibliometric analysis

Analysis of the descriptive statistics of the key metadata, the time series of publications, and the sources of local impact, author impact, leading institutions, leading countries, and publication performance was performed using the bibliometrix tool in R for Windows. VOSviewer (28, 29) software was utilized for visual analysis of the co-authorship networks, the co-occurrence of reported keywords, and the citation and co-citation maps based on bibliographical data. Thematic map visualization and evaluation were conducted using bibliometrix to carry out conceptual network analysis, factorial analysis, and social network analysis. Furthermore, collaboration network analysis was performed and country collaboration maps were analyzed using RStudio software (version 4.4.3) and bibliometrix, an R package and online analysis platform. GraphPad Prism 9 (version 9.2.0; GraphPad Software LLC, San Diego, CA, USA) and OriginPro 2019 (v9.6) were used for inference statistical analysis and for the correlations among the study variables. A *p*-value ≤0.05 was considered statistically significant.

## Results

### Performance analysis of the calcimimetic-associated publications

A total of 4,896 documents were identified after merging the two databases. There were 3,500 documents identified and included in the bibliometric analysis after removal of duplicate publications.

**Figure 2:**
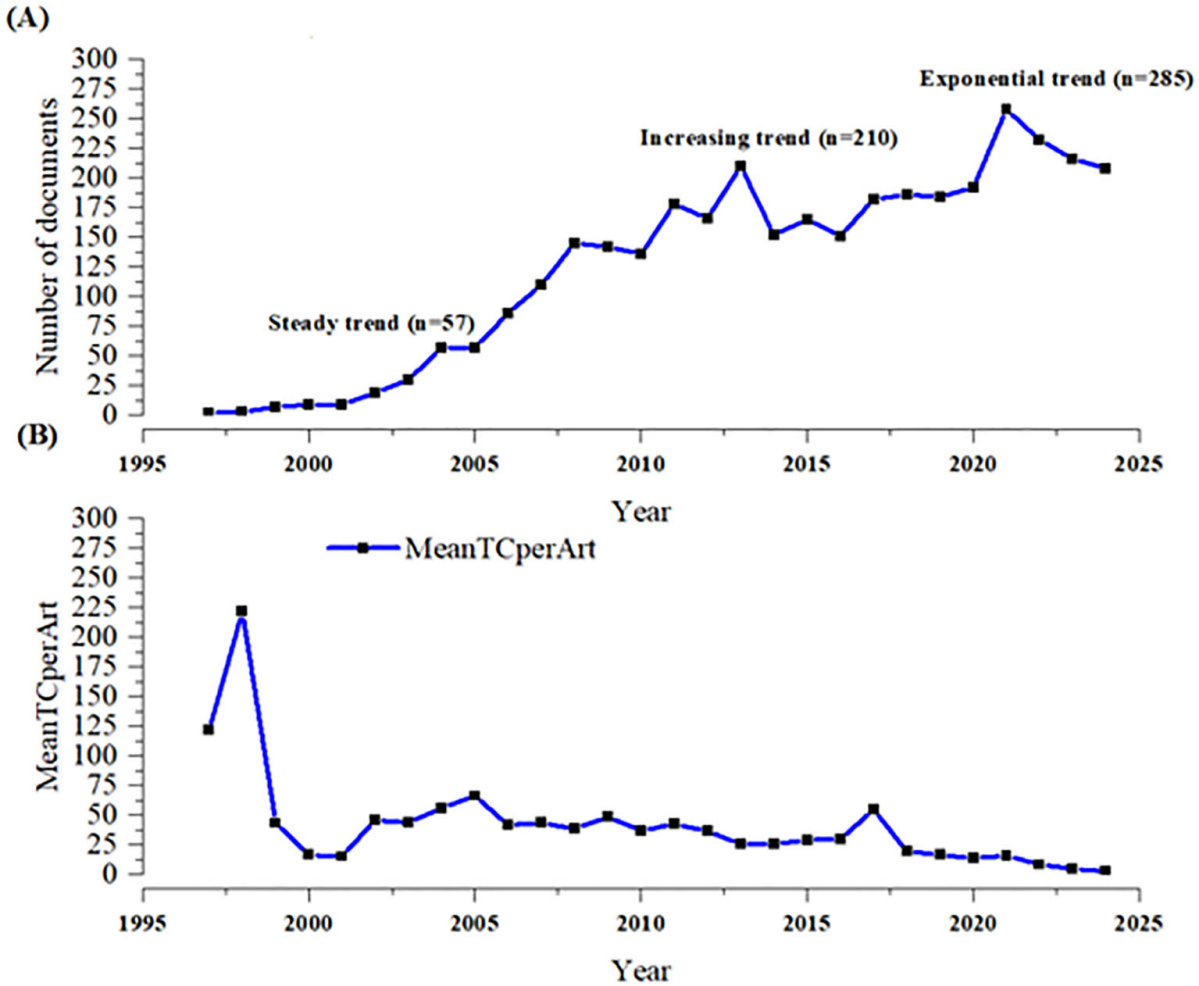
Annual number of publications **(A)** and citations **(B)** on the topic of Calcimimetics using data merged from WOS and Scopus databases (1997 to 2025) Source: Generated by the authors.

**Figure 2** shows the annual number of publications and citations of calcimimetic-associated publications from the WoS and Scopus databases included in the analysis, from 1997 to 2025. There was a continuous trend in scientific output between 2004 and 2010 (a steady trend). From 2010 to 2013 onwards, the evolution showed an exponential growth in calcimimetic-associated publications (increasing and exponential trend in 2021), corresponding with specific studies on calcimimetics from both WoS and Scopus (**Figure 2A**). The mean of the total citations per article (MeanTCperArt) showed an increase in 1998, with a total of 226 MeanTCperArt, as displayed in **Figure 2B**.

**Table 2** shows the descriptive summary statistics of the published articles on calcimimetics, including the year, sources, annual growth, document content, authors, and document types during the period 1997–2024. There were 3,500 documents including 2,683 (76.6%) articles and 817 (23.34%) reviews, published in 1,108 sources by 12,439 authors, with 287 single-authored documents and with 10.23% international co-authorships. The number of published documents per year rapidly increased, with 285 associated articles published in 2021. There was a significant negative correlation between the number of articles published and the total citations per year *(r* = −0.95, *p* = 0.0001).

**Table 2:** Descriptive summary statistics.

Description	Results	Description	Results
Main data profile on calcimimetics		Authors	
Time span	1997–2024	Authors	12,439
Sources (journals, books, etc.)	1,108	Authors of single-authored documents	210
Documents	3,500	Author collaboration	
Annual growth rate (%)	5.08	Single-authored documents	287
Document average age	9.61	Co-authors per document	5.9
Average citations per document	27.29	International co-authorships (%)	10.23
Document content		Document type	
KeyWords Plus (ID)	16,644	Article, *n* (%)	2683 (76.6)
Authors’ keywords (DE)	4,475	Review, *n* (%)	817 (23.34)

### Authorship analysis

**Figure 3:**
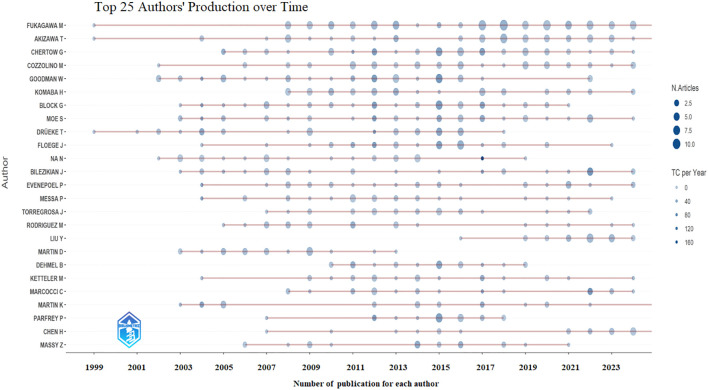
Top 25 productive authors over time based on number of articles and total citations (TCs) per year.

A total of 1,243 authors contributed to the global research on calcimimetics. The papers published by W. Goodman received a high index, followed by those of G. Block, M. Fukagawa, and G. Chertow (**Table 3**). The annual trends of the top 25 productive authors over time based on the number of articles and the total citations per year (TCperYear) are displayed in **Figure 3**. There was a significant positive correlation between the number of articles published by authors and the *h*_index (*r* = 0.9243, *p* < 0.0001), followed by the *g*_index (*r* = 0.9844, *p* < 0.0001), the *m*_index (*r* = 03718, *p* < 0.0001), and the total number of citations (TNC) (*r* = 0.4722, *p* < 0.0001).

**Table 3:** Top 10 authors of calcimimetic-associated publications from the Web of Science (WoS) and Scopus databases based on the *h*_index (1997–2024).

Author (*N* = 1,243)	*h*_index	*g*_index	TNC	TNP	PY_start
Goodman, W.	27	45	4,768	45	2002–2022
Block, G.	26	40	4,084	40	2003–2021
Fukagawa, M.	25	48	2,658	97	1999–2024
Chertow, G.	22	50	3,740	50	2005–2024
Moe, S.	21	38	3,976	38	2003–2024
Bilezikian, J.	20	28	2,465	28	2003
Drüeke, T.	20	34	3,471	34	1999–2018
Evenepoel, P.	20	27	1,991	27	2004–2024
Floege, J.	19	31	2,922	31	2004–2023
Komaba, H.	19	38	1,522	41	2008–2024

*TNC*, total number of citations; *TNP*, total number of publications; *PY_start*, publication year start.

### Top 10 journals with published articles on calcimimetics

**Figure 4:**
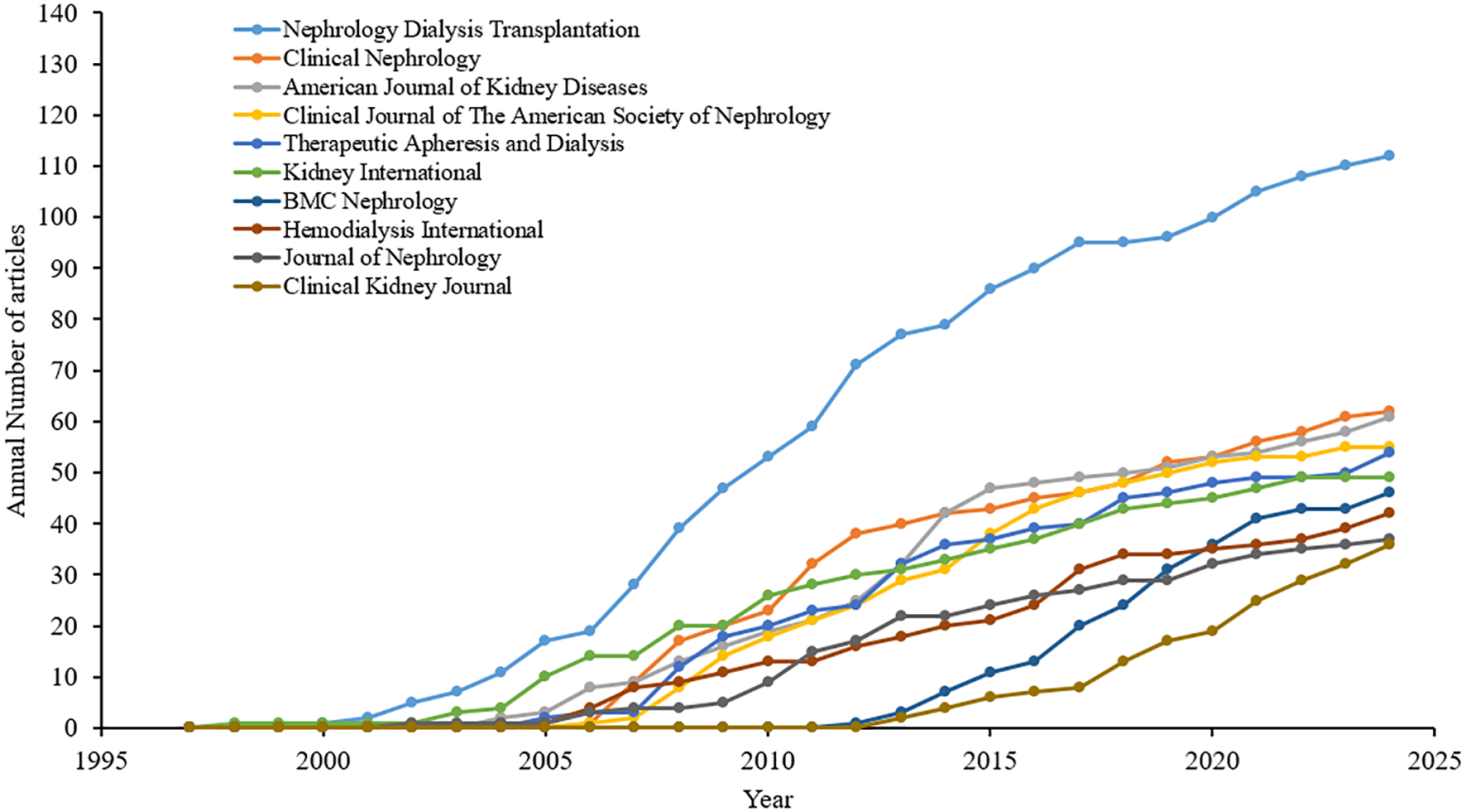
Chronological analysis of the top 10 publication sources of calcimimetic-associated reports from the Web of Science (WoS) and Scopus databases according to cumulative publications (1997–2024).

There were 1,108 journals identified to have contributed 3,500 calcimimetic-associated publications from the WoS and Scopus databases. Of the top 10 sources, Nephrology Dialysis Transplantation is the leading resource. In 1998, Kidney International published the first article on calcimimetics. Nephrology Dialysis Transplantation showed an upward trend since early 2000 up to 2024 (**Figure 4**). After 2005, the number of publications on calcimimetics in each source began to increase rapidly. The most notable increase according to the cumulative publications was observed in the “Kidney International” journal, followed by the “American Journal of Kidney Diseases.” Nephrology Dialysis Transplantation showed a higher growth rate with 112 published articles, with a citation score of 5,568 compared with the top 10 journals (**Table 4**). The chronological analysis of the top 10 publication sources on calcimimetic-associated publications is presented in **Figure 4**. There was a significant positive correlation between the number of articles and the *h*_index (*r* = 0.9573, *p* < 0.0001), the *g*_index (*r* = 0.6990, *p* < 0.0001), the *m*_index (*r* = 00.6990, *p* < 0.0001), and the TNC (*r* = 0.6669, *p* < 0.0001).

**Table 4:** Top 10 journals that published calcimimetic-associated articles from the Web of Science (WoS) and Scopus databases by number of local citations and *h*_index (1997–2024).

Source (*n* = 1,108)	*h*_index	*g*_index	TNC	TNP	PY_start	Orion	JIF (2024)
Nephrology Dialysis Transplantation	43	71	5,568	112	2000	England	4.8
Clinical Journal of the American Society of Nephrology	34	56	4,251	56	2006	England	4.8
Kidney International	31	49	3,994	49	1998	USA	14.8
American Journal of Kidney Diseases	29	50	2,544	61	2004	USA	9.4
Journal of Clinical Endocrinology & Metabolism	22	35	2,033	35	2003	USA	5.0
Journal of The American Society of Nephrology	21	31	2,481	31	2002	USA	10.3
Clinical Nephrology	16	29	983	63	2006	German	1.1
Pediatric Nephrology	15	23	596	34	2003	USA	2.6
Plos One	15	26	700	34	2011	USA	2.9
Therapeutic Apheresis and Dialysis	15	30	994	54	2005	Japan	1.5

*TNC*, total number of citations; *TNP*, total number of publications; *PY_start*, publication year start.

### Analysis of the top 25 relevant affiliations or institution influence (1997–2024)

**Figure 5:**
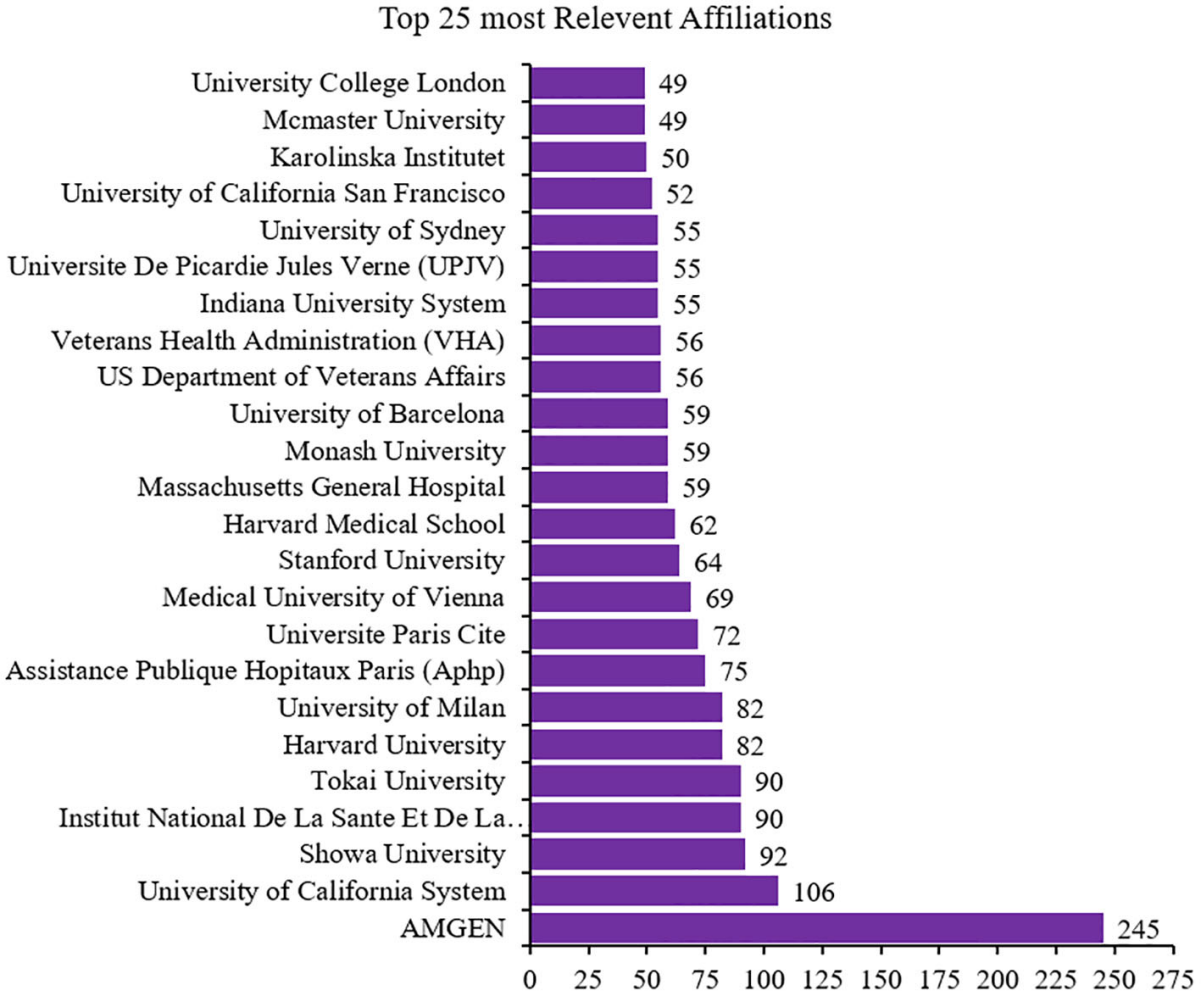
Top 25 most relevant affiliations ranked by total number of articles (*N* = 3,931).

Of the 3,931 institutions in the reports, Amgen had greater influence, with a contribution of 245 articles. The University of California System ranked second in institutions with more influence on the dynamics of publishing articles on calcimimetics (**Figure 5**).

### Corresponding authors’ countries and national and international analyses

Analysis of global calcimimetics publications revealed contributions from 75 countries, resulting in 1,243 instances of contributions of authors globally, with 10.23% international co-authorships (**Tables 1**, **5**). **Table 5** details the top 10 countries that contributed to calcimimetic research productivity. The USA emerged as the most prolific contributor, with 841 total publications including (SCP=759) single-country publications, (MCP=82) and multi-country publications (MCP=8). This was followed by Japan, with 356 total publications comprising 337 SCP and 19 MCP. In addition, China ranked among the leading contributors, with 7% total number of publications (TNP = 229), among them 215 SCP and 14 MCP. The most highly cited articles are presented in **Table 5**.

**Table 5:** Corresponding author’s countries on calcimimetics and most cited countries associated publications from WoS and Scopus databases (1997 to 2024).

Country (*N* = 75)	Articles	Articles (%)	SCP	MCP	MCP (%)	Country	TNC
USA	841	24	759	82	9.8	USA	33,782
Japan	356	10.2	337	19	5.3	Japan	6,659
China	229	6.5	215	14	6.1	United Kingdom	5,151
Italy	214	6.1	187	27	12.6	France	4,847
United Kingdom	157	4.5	133	24	15.3	Italy	4,811
Spain	146	4.2	122	24	16.4	Australia	4,223
France	142	4.1	114	28	19.7	Spain	3,676
Germany	120	3.4	97	23	19.2	Germany	3,657
Canada	94	2.7	78	16	17	Denmark	3,313
Australia	91	2.6	83	8	8.8	Canada	2,950

*TNC*, total number of citations; *SCP*, single-country publications; *MCP*, multiple-country publications.

**Table 6** shows the top 10 most cited calcimimetic-associated publications from the WoS and Scopus databases.

**Table 6:** Top 10 most cited calcimimetic-associated publications from the Web of Science (WoS) and Scopus databases (1997–2024).

Paper	DOI	Total citations
Block, G., 2004, New Engl J Med	10.1056/NEJMoa031633	853
Chertow, G., 2012, New Engl J Med	10.1056/NEJMoa1205624	687
Fraser. W., 2009, Lancet	10.1016/S0140-6736(09)60507-9	677
Nemeth, E., 1998, Proc Natl Acad Sci USA	10.1073/pnas.95.7.4040	526
Raggi, P., 2011, Nephrol Dial Transpl	10.1093/ndt/gfq725	426
Cunningham, J., 2011, Clin J Am Soc Nephro	10.2215/CJN.06040710	421
Cunningham, J., 2005, Kidney Int	10.1111/j.1523-1755.2005. 00596.x	384
Khan, A., 2017, Osteoporosis Int	10.1007/s00198-016-3716-2	367
Chong, W., 2011, Endocr-Relat Cancer	10.1530/ERC-11-0006	346
Ortiz, A., 2014, Lancet	10.1016/S0140-6736(14)60384-6	344

### Keyword analysis

**Figure 6:**
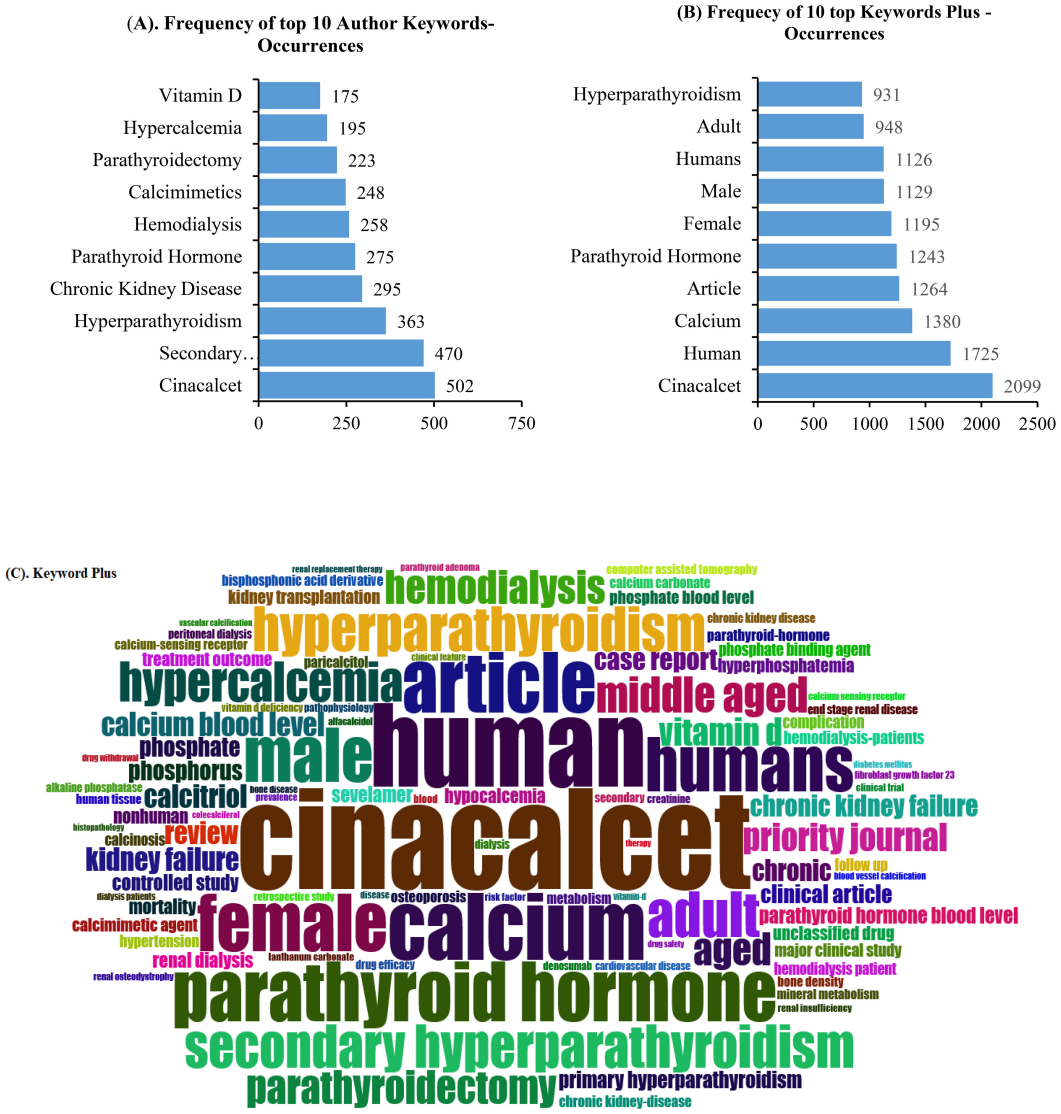
Top 10 most frequent authors’ keyword **(A)** and KeyWords Plus **(B)** occurrences. **(C)** Visualization of the top 100 most frequent KeyWords Plus.

The top 10 keywords based on the authors’ keywords and KeyWords Plus in terms of frequency distribution are presented in **Figure 6**. Visualization of the authors’ keywords showed that cinacalcet, secondary hyperparathyroidism, hyperparathyroidism, chronic kidney disease, parathyroid hormone, hemodialysis, calcimimetics, parathyroidectomy, hypercalcemia, and vitamin D, among others, were the most common topics covered (**Figure 6A**). However, the top 10 most frequently presented KeyWords Plus were cinacalcet, human, calcium, article, parathyroid hormone, female, male, humans, adult, and hyperparathyroidism, among other keywords. The top 100 KeyWords Plus occurrences are displayed in **Figure 6C**.

### Thematic analysis

**Figure 7:**
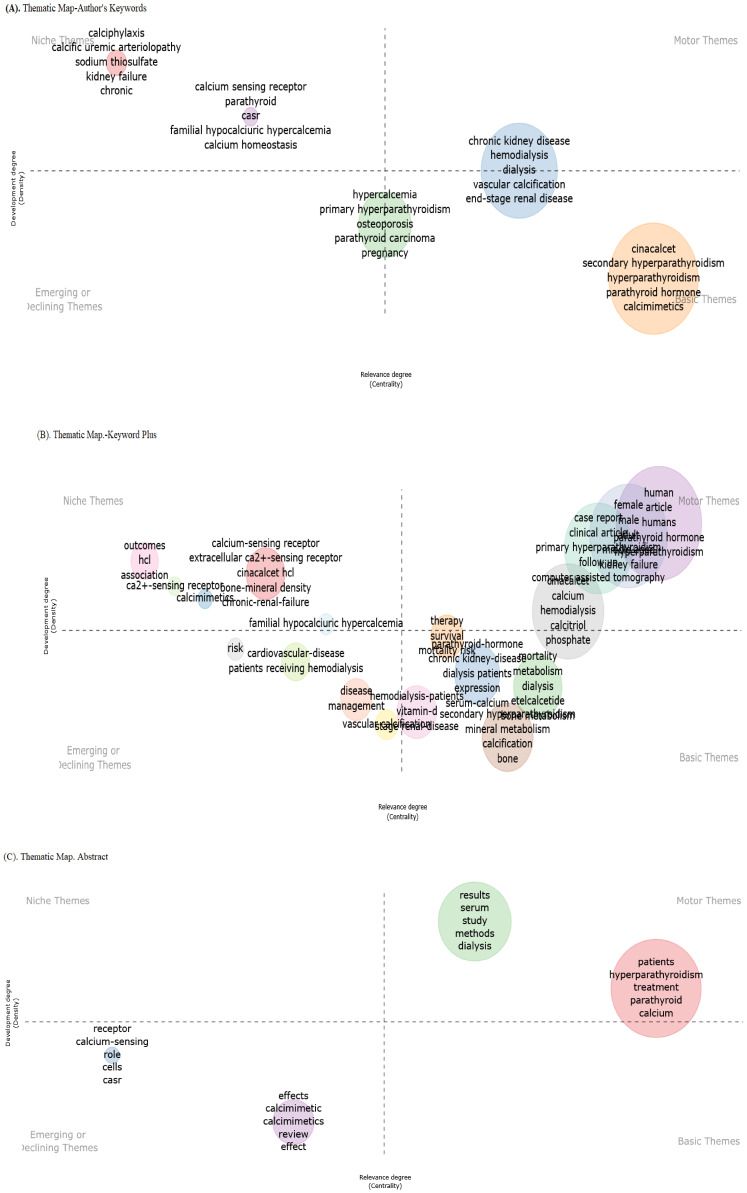
Thematic map of the authors’ keywords **(A)**, KeyWords Plus **(B)**, and abstracts **(C)**.

The thematic map of the authors’ keywords revealed four distinct clusters of research themes related to calcium metabolism and kidney disease, indicating interdisciplinary connections. The key observations reported cluster “chronic kidney disease (CKD) & mineral disorders” in basic and motor themes, followed by “calcium homeostasis & genetic disorders” in niche themes, which included highlights of the genetic and molecular mechanisms of calcium regulation [familial hypocalciuric hypercalcemia (FHH) as a niche area]. The “hypercalcemia” cluster linked hypercalcemic states to bone pathologies, located in the emerging and basic themes. “Chronic kidney disease” was the most dominant research, and “vascular calcification” was a critical sub-theme (**Figure 7A**).

The thematic KeyWords Plus analysis in **Figure 7B** identified three key domains, including pharmacological targets (CaSR and cinacalcet) reported in niche themes and central CKD-MBD pathway (HD, vascular calcification, and vitamin D) cases as reported in emerging/declining themes. The small association between “diabetes” and “mineral metabolism” (despite shared CKD complications) suggests a critical research gap.

**Figure 7C** illustrates a thematic clustering process that used abstract words, likely from a biomedical or pharmacological study, focusing on keywords related to calcium sensing and receptor modulation. The structure suggests an emerging thematic analysis, where the dominant topics were grouped hierarchically or by relevance. These findings suggest that “calcium-sensing receptors” and “effects calcimimetic” are major research subjects.

### Thematic evolution

In **Figure 7**, the context of thematic evaluation on calcimimetic-associated publications identified patterns or themes in data from qualitative research using the Walktrap algorithm, with 250 number of words with minimum five-word frequency (per thousand documents) parameters used to identify clusters of related themes.

**Figure 8:**
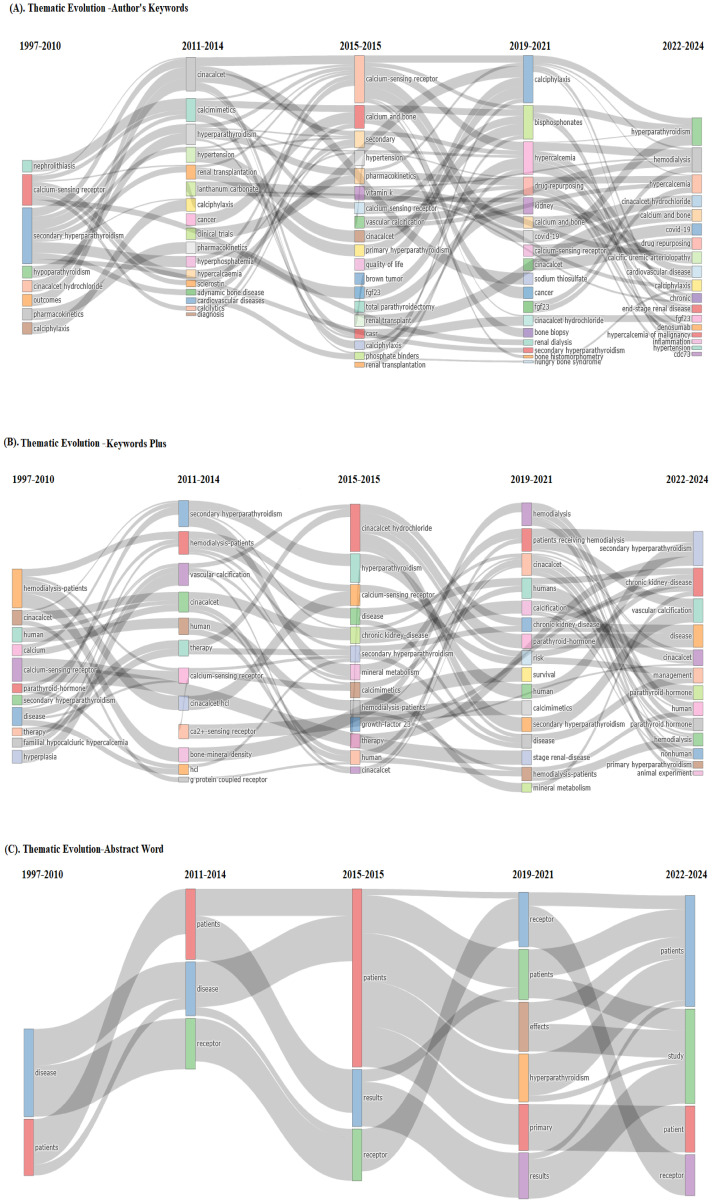
Existing and emerging themes on calcimimetic-associated publications from the Web of Science (WoS) and Scopus databases using thematic evolution within four time slices of the authors’ keywords **(A)**, KeyWords Plus **(B)**, and title words **(C)**. Source: Biblioshiny, based on the Scopus dataset associated with calcimimetic-related publications from WoS and Scopus from 1997 to 2024.

Analysis of the existing and emerging themes on calcimimetic-associated publications using thematic evolution within four time slices, i.e., basic and motor themes (1997–2010), niche and motor themes (2011–2014), emerging themes (2015–2015), and emerging and future motor themes (2019–2024), of the authors’ keywords, KeyWords Plus, and title words (**Figures 8A–C**, respectively) suggests potential future research directions or emerging areas of interest within calcimimetic-associated publications globally, as well as further comprehensive details for understanding the role of CaSR in calcium homeostasis, the pharmacodynamics of cinacalcet and etelcalcetide, and core research areas. Moreover, the trends showed clinical applications in hyperparathyroidism such as in primary and secondary hyperparathyroidism and highlighted the area of adverse effects and safety profile, including cardiovascular risks and hypocalcemia and CKD.

The thematic evolution analysis showed that the research on calcimimetics has evolved from fundamental studies on CaSR mechanisms and hyperparathyroidism treatment to new areas such as artificial intelligence (AI)-based drug response predictions and novel calcimimetic formulations. Emerging themes in the phase of authors’ keywords suggest future research directions focusing on calciphylaxis, bisphosphonates, hypercalcemia, drug repurposing, kidney, calcium and bone, COVID-19, CaSR, cinacalcet, sodium thiosulfate, cancer, fgf23, cinacalcet hydrochloride, SHPT, bone histomorphometry, hungry bone syndrome, calcic uremic arteriolopathy, cardiovascular disease, calciphylaxis, chronic, denosumab, hypercalcemia of malignancy, inflammation, hypertension, and cdc73, which have evolved significantly based on the publications indexed in WoS and Scopus (**Figure 8A**).

Emerging themes in the phase of KeyWords Plus showed new and growing topics by scholars including research focused on SHPT, CKD, vascular calcification, disease, cinacalcet, management, parathyroid, PTH, hemodialysis, primary hyperparathyroidism, and animal experiment (**Figure 8B**).

Emerging themes in the phase of the title keywords, such as receptor, patients, effect, hyperparathyroidism, primary, results, and study, were more precise and directly highlighted the study’s main contribution in calcimimetic-associated publications (**Figure 8C**).

### Social structure: collaboration network

**Figure 9:**
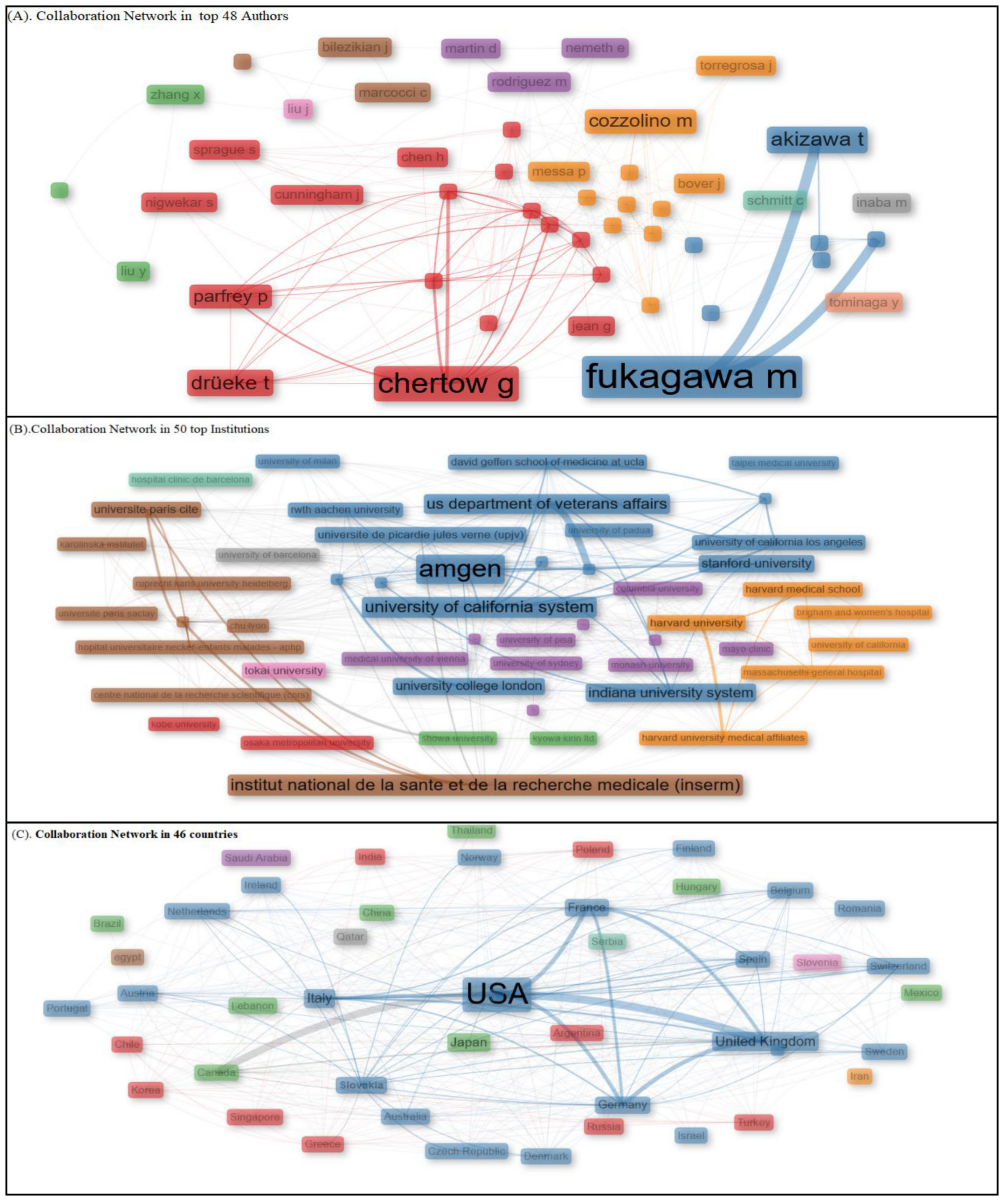
Collaboration Network analysis between top 48 authors, 50 institution, and 46 countries. Using Social Structure.

Collaboration between authors is a key factor in scientific research that highly contributes to influencing knowledge dissemination, further helping scholars identify leading researchers, research groups, and global collaborations working on calcimimetic-associated publications (**Figure 9A**, **Table 2**).

#### Collaboration between institutions

**Figure 9B** displays the collaboration network based on the betweenness centrality scores, with the top institutions grouped into nine distinct clusters based on their influence within the network. Cluster 1 included higher education institutions with moderate betweenness centrality values, such as Kobe University (1.572) and Osaka Metropolitan University (7.8). Cluster 2 consisted of institutions with high betweenness centrality, showcasing significant influence in global research productivity related to calcimimetic-associated publications. These included Amgen (139.842), University of California System (39.694), University of Milan (5.867), Stanford University (28.049), U.S. Department of Veterans Affairs (33.782), Veterans Health Administration (33.782), Indiana University System (24.948), Université de Picardie Jules Verne (9.177), University of California San Francisco (6.26), University College London (15.396), University of California Los Angeles (0.976), University of London (9.177), Taipei Medical University (0.0), David Geffen School of Medicine at UCLA (0.698), University of California Los Angeles Medical Center (0.698), and Indiana University Bloomington (9.92). Cluster 3 included institutions with lower betweenness centrality values, such as RWTH Aachen University (5.773), University of Padua (0.293), and Showa University (0.91). Cluster 4 comprised a variety of institutions with varied betweenness values, including Kyowa Kirin Ltd. (0.125), Medical University of Vienna (1.104), Monash University (1.263), University of Sydney (11.491), McMaster University (8.692), University of Toronto (7.494), Columbia University (11.703), University of Copenhagen (11.537), University of Pisa (12.238), University of Oxford (9.553), and Mayo Clinic (0.455). Cluster 5 included prestigious institutions with strong betweenness centrality values, such as Harvard University (76.543), Harvard Medical School (7.463), Massachusetts General Hospital (6.398), Harvard University Medical Affiliates (6.368), Brigham and Women’s Hospital (0.621), and University of California (1.712). Cluster 6 included research institutions with significant betweenness values, such as Institute National de la Santé et de la Recherche Médicale (88.026), Assistance Publique Hôpitaux Paris (21.156), Université Paris Cité (8.243), Karolinska Institutet (0.128), Ruprecht Karls University Heidelberg (7.787), Université Paris Saclay (4.971), Hôpital Universitaire Necker-Enfants Malades (0.234), and Centre National de la Recherche Scientifique CHU Lyon (4.238). Cluster 7 consisted of Tokai University, with significant betweenness centrality (33.734). Cluster 8 included University of Barcelona, which had notable betweenness centrality (14.826). In cluster 9 was a hospital with low betweenness centrality (0.0).

The collaboration network highlighted the varying levels of centrality, with some institutions or universities such as Amgen, Institute National de la Sante et de la Recherché Medical, Harvard University, University of California System, US Department of Veterans Affairs, Veterans Health Administration, Tokai University, Assistance Publique Hopitaux Paris, Stanford University, Indiana University System, and University Paris cited. This collaboration network plays a pivotal role in calcimimetic-associated research. Others showed minor interconnectedness in the global academic and research landscape.

**Figure 9B** displays the collaboration network based on the betweenness centrality scores, with the top institutions grouped into nine distinguished clusters based on their influence in the network. Cluster 1 included institutions of higher education with moderate betweenness centrality values, such as Kobe University (1.572) and Osaka Metropolitan University (7.8). Cluster 2 comprised institutions such as Amgen (139.842), followed by the University of California System (39.694), University of Milan (5.867), Stanford University (28.049), U.S. Department of Veterans Affairs (33.782), Veterans Health Administration (33.782), Indiana University System (24.948), Université de Picardie Jules Verne (9.177), University of California San Francisco (6.26), University College London (15.396), University of California Los Angeles (0.976), University of London (9.177), Taipei Medical University (0.0), David Geffen School of Medicine at UCLA (0.698), University of California Los Angeles Medical Center (0.698), and Indiana University Bloomington (9.92), characterized by high betweenness centrality and showed a significant level of influence in the global research productivity on calcimimetic-associated publications. Cluster 3, which is characterized by institutions with lower betweenness centrality, included RWTH Aachen University (5.773), University of Padua (0.293), and Showa University (0.91). Cluster 4 included Kyowa Kirin Ltd. (0.125), followed by Medical University of Vienna (1.104), Monash University (1.263), University of Sydney (11.491), McMaster University (8.692), University of Toronto (7.494), Columbia University (11.703), University of Copenhagen (11.537), University of Pisa (12.238), University of Oxford (9.553), and Mayo Clinic (0.455), universities and institutions with varied betweenness values. Cluster 5 included prestigious institutions such as Harvard University (76.543), Harvard Medical School (7.463), Massachusetts General Hospital (6.398), Harvard University Medical Affiliates (6.368), Brigham and Women’s Hospital (0.621), and University of California (1.712), showcasing strong betweenness centrality. Cluster 6 included research institutions with significant betweenness values: Institute National de la Santé et de la Recherche Médicale (88.026), Assistance Publique Hôpitaux Paris (21.156), Université Paris Cité (8.243), Karolinska Institutet (0.128), Ruprecht Karls University Heidelberg (7.787), Université Paris Saclay (4.971), Hôpital Universitaire Necker-Enfants Malades (0.234), and Centre National de la Recherche Scientifique CHU Lyon (4.238). Cluster 7 included Tokai University, with significant betweenness centrality (33.734). In cluster 8 is the University of Barcelona, with notable betweenness centrality (14.826). A hospital with a low betweenness centrality (0.0) is included in cluster 9, as shown in **Figure 9B**.

#### Collaboration between countries

**Figure 9C** shows the network collaboration among the top 46 countries that contributed to calcimimetic-associated publications, highlighting their varying levels of centrality and influence within the network based on their betweenness centrality scores. The figure is divided into clusters based on the reported betweenness values, which classified the countries into groups with different levels of influence. The analysis showed cluster 1 to consist of countries such as India (0.588), Poland (4.131), Turkey (0.567), Greece (4.083), Korea (0.729), Singapore (0.146), Chile (0.429), Russia (0.103), and Argentina (0.0) with moderate-to-low betweenness values, demonstrating their less central roles in the network, followed by cluster 2 including countries such as the USA (178.394), United Kingdom (182.677), Italy (96.928), France (33.583), Spain (15.057), Germany (78.884), Australia (not specified), Austria (2.488), Netherlands (6.977), Belgium (7.95), Switzerland (8.047), Sweden (8.254), Denmark (7.698), Portugal (2.83), Finland (0.132), Romania (0.213), Israel (0.0), Czech Republic (0.063), Ireland (0.098), Norway (0.114), New Zealand (0.048), Slovakia (0.063), and Macedonia (0.0) that exhibited high betweenness values, indicating strong influence and centrality in the network. Cluster 3 represented Japan (0.361), China (1.107), Canada (18.897), Brazil (0.0), Thailand (0.0), Mexico (0.0), Hungary (0.0), and Lebanon (0.0), which signified moderate influence in the network due to the moderate betweenness scores reported. Cluster 4 only included Saudi Arabia, which had a betweenness value of 0.0, indicating a very low level of influence in scientific collaboration reported in calcimimetic-associated publications. Cluster 5 included Iran, with a betweenness value of 44, indicating a substantial impact within the network on scientific collaboration globally. Cluster 6 included Egypt, with a betweenness value of 1.285, reflecting moderate influence within the network. In cluster 7 is Slovenia, the only country in this cluster, which had a betweenness value of 0.0, indicating very low centrality. Qatar is in cluster 8, with a betweenness value of 0.0, indicating minimal influence in scientific collaboration between countries in the network. Serbia is the only country in cluster 9, with a betweenness value of 0.0, indicating limited connection and influence in the network (**Figure 9C**).

## Discussion

Since its introduction into the market, various quantitative systematic analyses have been published with regard to the use of calcimimetics. However, only a few qualitative analyses have been conducted to explore the literature on these issues. This bibliometric analysis provided an overview of the scientific publications, identified emerging trends and research topics on the use of calcimimetics, analyzed research collaborations, and highlighted influential research groups and collaborative clusters, as well as previous studies in different scientific fields. It also helps lay the groundwork for new or emerging aspects of topics within the area of calcimimetic research. The citation analysis of the published articles determined the most highly cited articles, emphasizing their great importance among scholars. The articles, published between 1997 and 2024, received 27.29 average citations per document and 10.23% international co-authorships. International co-authorships are widely encouraged by research funders in the belief that these are beneficial to scientific progress (30, 31).

M. Fukagawa from Tokai University, Japan, was the most prolific and active author, with 97 articles, total citation counts of 2,658, and an *h*_index of 25. M. Fukagawa also had the longest duration of research activity on calcimimetics (1999–2024). However, it is J. Bilezikian from Columbia University who had the highest number of publications calcimimetics per one year (*n* = 28) in 2003.

W. Goodman had the highest *h*_index (27), with 45 publications and 4,768 TNC. The *h*_index is an author-level metric that measures both the productivity and citation impact of a publication.

However, G. Chertow from Stanford University had the highest *g*_index (50), with 50 TNP and 3,740 citations. The high *g*_index reflects the depth of the impact of the articles published by this author, giving greater weight to highly cited papers. However, this author had fewer published articles than M. Fukagawa from Tokai University, Japan.

Based on research evidence, the most highly cited papers were published by G. Block. in 2004 in the New Engl J Med (853 citations), which explored the use of cinacalcet for SHPT in patients receiving HD. The study concluded that cinacalcet lowers the PTH levels and improves the calcium–phosphorus homeostasis in patients with uncontrolled SHPT receiving HD (32).

Among the hugely popular topics and highly cited articles published by Glenn M. Chertow in 2012, also in the New Engl J Med (with 687 citations), is the EVOLVE trial, which investigated the effect of cinacalcet on cardiovascular disease in patients undergoing dialysis. The results of cinacalcet hydrochloride therapy for lowering cardiovascular events (EVOLVE) trial were inconclusive, and the study failed to show any statistically significant effect on its primary outcome, a composite of cardiovascular events and death. However, predefined secondary and *post-hoc* analyses of the EVOLVE trial suggested beneficial effects both in general and in subgroups. As a consequence of these mixed results, it was proposed that while cinacalcet could be used as one of several drugs to improve the achievement of biochemical control in CKD-related MBD as recommended by international guidelines, it should not be used with the purpose of decreasing the cardiovascular outcomes and/or improving survival (33).

After the EVOLVE trial, the focus moved to the impact of cinacalcet on hard clinical outcomes to assess its effects on cardiovascular calcification and the risk of cardiovascular events and mortality (34–36).

Two highly influential journals that significantly advanced research on calcimimetics were Nephrology Dialysis Transplantation and the Clinical Journal of the American Society of Nephrology, which received 5,568 and 4,251 citations, respectively. These most highly cited publications played a crucial role in broadening scholarly evidence with regard to the application of calcimimetics.

Most of the publications on calcimimetics were from the USA, Japan, and China. The USA was the most productive in single-country production with 841 articles, followed by Japan and China. In the top country categories, there were massive research contributions from developed countries. The findings of this retrospective study highlight the geographic distribution of the research contributions to the global research on calcimimetics. Notably, North America, Europe, and Asia emerged as the leading regions across all examined categories, including authorship, institutional affiliation, and funding sources in the publications indexed by WoS and Scopus. Calcimimetic documents received significantly more citations in countries such as the USA, Spain, and Japan compared with others, leading to improved research outcomes and journal metrics for both the authors and journals involved.

In contrast, regions with lower levels of contribution, Africa being one of them, require substantial support to enhance their research productivity, particularly in resource-limited settings. To enhance the research progress in other countries, we recommend breaking down barriers, encouraging both national and international fostering and both national and international collaborations, and fostering exchanges between various nationals and international organizations.

The large number of scientific institutions or affiliations involved (3,931) provided evidence that research institutions play a crucial role in driving the research productivity on calcimimetic-associated publications from the WoS and Scopus databases over the past years. Of these institutions, Amgen had greater influence with contributions in 245 articles, making it more likely to foster outstanding researchers and to make exceptional contributions in relevant calcimimetic-associated publications. This could be explained by Amgen being the first company to license cinacalcet. Cinacalcet is the first calcimimetic approved for clinical use under the brand name Mimpara^®^ in 1996 in Europe. In March 2004, the FDA also approved cinacalcet, which is marketed as Sensipar^®^ in the USA. Cinacalcet remains the most prescribed calcimimetic drug (37).

A significant correlation between the number of articles [the journal impact factor (JIF, 2023)] and the *h*_index was observed for the journals that published articles on calcimimetics from 1997 to 2024. For example, Nephrology Dialysis Transplantation published 112 articles (*h*_index = 43), followed by the Clinical Journal of the American Society of Nephrology and Kidney International [*n* = 56 (*h*_index = 34) and *n* = 49 (*h*_index = 31), respectively]. The Journal of the American Society of Nephrology had the least number of articles published [*n* = 31 (*h*_index = 21].

Across all branches of science in calcimimetic research published in the WoS and Scopus databases, all of the top 10 most cited articles were published in 1998–2011. Overall, these articles received more than 24,144 citations from global researchers.

“Cinacalcet” was the most explored calcimimetics by authors according to the keyword and KeyWords Plus analysis, in addition to PTH and hyperparathyroidism. This finding reflects the authors’ concern about the effect of calcimimetics on the management of hyperparathyroidism, which can result in extremely serious complications particularly for patients with CKD.

However, the research gap concerning the clinical usefulness of calcimimetics remains unaddressed, with the keyword and KeyWords Plus analysis failing to highlight this critical issue—particularly in light of the disappointing results from the EVOLVE trial in 2012 and the subsequent controversy.

The thematic evolution analysis showed that the research on calcimimetics evolved from fundamental studies on CaSR mechanisms and hyperparathyroidism treatment to new areas such as AI-based drug response predictions and novel calcimimetic formulations in light of the evidenced high adverse effect profile of many calcimimetics. Emerging themes in the phase of authors’ keywords suggest future research directions focusing on calciphylaxis, bisphosphonates, hypercalcemia, drug repurposing, kidney, and cardiovascular disease. Emerging themes in the phase of KeyWords Plus show new and growing topics by scholars such as research focused on SHPT, CKD, vascular calcification, and primary hyperparathyroidism.

## Conclusion and future research directions

The results of this study offer a summary of the global landscape, key research areas, and possible future directions in calcimimetic research. This information can assist researchers in exploring the knowledge structure and understanding future trends in calcimimetic research. Bibliometric studies such as this are valuable for researchers and policymakers, helping them evaluate and understand the knowledge structure and guide future development trends in calcimimetic research.

The strengths of this study include its provision of a qualitative and thematic analysis of the most comprehensive literature on calcimimetic research globally, highlighting how far scientific research and scholars have contributed over the past decades. We also recognize certain limitations of our study. We relied solely on the WoS and Scopus databases for identifying publications, which meant that studies indexed in other databases (e.g., PubMed, Medline, and Google Scholar) may have been overlooked. However, we believe that using the Science Citation Index Expanded of the WoS and the Scopus databases offers a strong foundation for the analysis of research performance in calcimimetics. In addition, we suggest that incorporating data from other sources would have introduced more diversity, leading to a more comprehensive bibliometrix analysis.

This study highlighted the extent of calcimimetic use and expanded the knowledge on the most prominent articles, authors, publishing journals, and countries. Support should be accorded to increase the publication and funding in low-resource settings with low publications. Furthermore, the collaboration analysis showed that there is a need for scientific health policies to promote international partnerships toward enhancing research infrastructure and to increase participation in global scientific collaborations. The aggregate response toward the collaboration issue can help in further understanding the global collaboration dynamics and improving the scientific cooperation between countries working in the field.

In terms of institution or university collaboration in global research productivity, there is a need for future research that could explore how these institutions’ varying centralities influence collaborative efforts and scientific advancements toward global calcimimetic-associated publications.

## Data Availability

The datasets presented in this study can be found in online repositories. The names of the repository/repositories and accession number(s) can be found below: https://www.scopus.com, https://www.webofscience.com/wos.
